# Health in old age – status quo, challenges, and opportunities

**DOI:** 10.25646/11668

**Published:** 2023-09-20

**Authors:** Janine Stein, Steffi G. Riedel-Heller

**Affiliations:** Leipzig University, Faculty of Medicine Institute of Social Medicine, Occupational Health and Public Health (ISAP), Germany

Health monitoring of the older society is of growing sociopolitical relevance for several reasons. In the course of current demographic developments, a significant increase in the number of older people can be expected in the coming decades. In particular, the very old (85+ years) belong to the fastest growing group. This is accompanied by increasing morbidity at the population level, with mental and neurodegenerative diseases in old age, such as dementia, being the most common [1]. The trend, that the need for care and nursing will increase in old age, is evident [2]. Against this background, the prevention and specific treatment of common diseases among older adults, especially dementias, represent a major challenge and an important task.

Current epidemiological data and research findings on the health situation of older people are indispensable for federal health reporting in order to explore prevention and treatment potentials and thus enable specific support. The epidemiological longitudinal study Gesundheit 65+ was launched to examine the health situation of old and very old people in Germany. In this issue, Fuchs et al. initially present the study and its objectives, contents and implementation. With Gesundheit 65+, a nationwide population-based health study with the target group of the German older population and a special focus on health limitations was realised for the first time to support the ongoing national health reporting. Based on this, Gaertner et al. present first study results of the baseline survey. In addition to the reported high level of life satisfaction, a number of indicators related to the living environment, activity and participation, as well as health functions were examined, and actual data were presented by means of prevalence calculations. Their findings provide an essential starting point for the derivation of policy and practice-related recommendations for action.

The review by Georges et al. provides a comprehensive overview of demographic developments, risk factors and care options for dementia in Germany. In old age, cognitive disorders and dementia are among the most common disorders and have serious impact on individuals and the community. Consequences include increasing dependence in daily life, a growing need for support and care, institutionalisation, functional limitations, mortality, need for care as well as high costs for the health care system. In the early stages of the disease, little support may be needed, which is often provided by partners or family as informal caregivers and people who give practical assistance. As dementia progresses, caregiver resources are often exceeded and formal care (such as domestic or day care) becomes necessary to ensure that care needs are met. According to the latest predictions by the German Federal Statistical Office and only because of aging, the number of people in need of care in Germany will increase from around 5.0 million at the end of 2021 to around 6.8 million in 2055 (by 37 %). Most of the increase by 2055 will be attributable to very old people aged 80 and older in need of long-term care [2].

While in Germany and worldwide the number of people with dementia will increase in the future, at the same time some factors hold an enormous potential for prevention. In this course, current research has identified potentially modifiable risk factors for dementia and investigated them in the context of interventions that address different risk factors simultaneously [3, 4]. Key factors include low education, hypertension, hearing impairment, smoking, obesity, depression, physical inactivity, and diabetes, as well as loneliness and social isolation in old age [5]. In a fact sheet, Wurm et al. present data on the prevalence of loneliness among older adults in Germany based on the German Ageing Survey (DEAS), a nationally representative cross-sectional and longitudinal survey of persons aged 40 years and older. Since loneliness and lack of social participation can significantly increase the risk of dementia, the observation of loneliness rates forms a substantial starting point for the initiation of preventive measures, not least against the background of the ongoing consequences of the COVID-19 pandemic [6].

Based on the German Aging Survey, Wurm et al. explore in another fact sheet the question of how many older persons in Germany hold an advance directive and what the determinants are. An advance directive, which contains the patient’s personal will regarding medical treatments and emergency situations, is of great importance in the context of care for older individuals. Regarding age effects, it has already been shown for very old GP patients aged 85 years and older that a substantial proportion have an advance directive and power of attorney for health care [7]. In contrast, this issue shows that there is room for improvement among younger old people.

Acknowledging the importance of health in old age, this issue of the Journal of Health Monitoring highlights the collaborative efforts needed to maintain and improve it. This is precisely why the United Nations has declared 2021 – 2030 the Decade of Healthy Aging [8].
